# Necrotizing Fasciitis of Eyelid in Children: A Rare Complication of Varicella Infection

**DOI:** 10.1002/ccr3.71334

**Published:** 2025-10-21

**Authors:** Govinda Bhandari, Himal Acharya, Richa Paudyal

**Affiliations:** ^1^ Institute of Medicine Maharajgunj Medical Campus Kathmandu Nepal; ^2^ B.P Koirala Lions Centre for Opthalmic Studies Kathmandu Nepal

**Keywords:** eyelid, necrotizing fasciitis, periorbital cellulitis, varicella

## Abstract

Necrotizing fasciitis (NF) of the eyelid is an extremely rare but severe complication of varicella zoster virus (VZV) infection in children. We present a case of an 8‐year‐old girl who developed necrotizing fasciitis of the left eyelid following varicella infection. The patient initially presented with fever, vesicular rashes, and swelling of the left peri‐orbital region. She was treated with oral acyclovir (400 mg five times daily for 7 days) and prednisolone (10 mg daily for 5 days) at a local medical store. Due to worsening symptoms, she was admitted to our center where intravenous antibiotics—Meropenem (40 mg/kg/dose every 8 h) and Vancomycin (15 mg/kg/dose every 6 h)—were administered for 21 days, followed by oral levofloxacin (250 mg once daily for 2 weeks). Surgical debridement of necrotic tissue was performed. Cultures revealed Coagulase‐negative Staphylococcus, Enterococcus, and later *Acinetobacter baumannii* . The patient responded well to treatment and showed healthy healing in follow‐up. This case emphasizes the importance of early surgical intervention, aggressive antibiotic therapy, and multidisciplinary care in managing rare but severe complications like periorbital necrotizing fasciitis secondary to varicella. Key clinical findings included progressive periorbital necrosis, culture results, and favorable response to combined medical and surgical therapy.

**Summary**

- Necrotizing fasciitis of the eyelid, though extremely rare, can complicate varicella infection in children.
- Early diagnosis, aggressive surgical intervention, and multidisciplinary care are crucial for managing this life‐threatening condition and preventing sight‐threatening complications like vision loss.
- Prompt recognition and treatment of necrotizing fasciitis in pediatric varicella cases are essential to improve outcomes and minimize morbidity.

## 1 Introduction

Varicella, which is also known as chickenpox, is a common childhood illness caused by varicella zoster virus and typically manifests with vesicular skin and mucosal rashes [1]. Chickenpox is generally milder in children than in older patients, but serious complications can still arise, particularly in immunosuppressed individuals and occasionally in healthy children [2]. The most common complication is secondary bacterial infection, such as abscess, cellulitis, necrotizing fasciitis (NF), and gangrene—terms often used interchangeably when discussing post‐varicella soft tissue infections, often caused by staphylococci or streptococci [3, 4]. In this case, we emphasize necrotizing fasciitis, a bacterial infection of the fascia, distinguished by rapid tissue necrosis and systemic toxicity. Gangrene, though overlapping in features, refers to extensive tissue death often involving ischemic or infective etiology [5]. Gangrene is a rare complication of varicella with a frequency of 0.05%–0.16%—a statistic derived from regional hospital data [6]. Periorbital NF is particularly rare due to the rich vascular supply of facial tissues [7]. The treatment approach included antiviral therapy with acyclovir to control the varicella infection, supportive care with broad‐spectrum intravenous antibiotics to manage secondary bacterial infection, and timely surgical debridement to remove necrotic tissue and prevent further spread of necrotizing fasciitis [6, 8]. Literature review revealed only three prior cases. This report outlines our diagnostic approach using clinical presentation, cultures, imaging, histopathology, and the Laboratory Risk Indicator for Necrotizing Fasciitis (LRINEC) score.

## 2 Case History/Examination

An 8‐year‐old girl presented to the emergency department of Tribhuvan University Teaching Hospital with a 5‐day history of fever and rashes and a 2‐day history of facial swelling, initially localized to the left upper eyelid. She complained of a vesicular rash over the back and chest region. While the patient had no confirmed history of direct exposure to an individual with active varicella or herpes zoster, there was a history of a similar rash and fever in her friends at school. After 3 days, she suddenly developed swelling only over the left upper eyelid; then the orbital swelling became more severe and later involved the whole face and neck too (Table 1). She has already visited some local medical stores for the rashes and swelling, where she was prescribed acyclovir and prednisolone. The rationale for prednisolone use remains unclear, as corticosteroids are not standard for varicella unless treating specific complications; this might reflect empirical treatment or local prescribing practices. The patient had no history of ibuprofen use, which is relevant as NSAIDs can exacerbate soft tissue infections. The symptoms didn't resolve and increased in severity, for which she had visited our center.

**TABLE 1:** Summary of clinical course and management.

Day	Clinical event	Management
1	Presentation with fever, rash, eyelid swelling	Started on oral acyclovir and prednisolone
3	Worsening symptoms	ICU admission, IV antibiotics, ophthalmology consult
4	Eyelid necrosis noted	Cultures sent, incision & drainage
5–26	IV antibiotics (Meropenem, Vancomycin)	Ongoing wound care
30	Re‐admission for worsening necrosis	Surgical debridement
31–45	Post‐op antibiotics (Flucloxacillin, Clindamycin)	Daily dressings
60	Recovery with granulation tissue	Discharged with follow‐up

**FIGURE 1:**
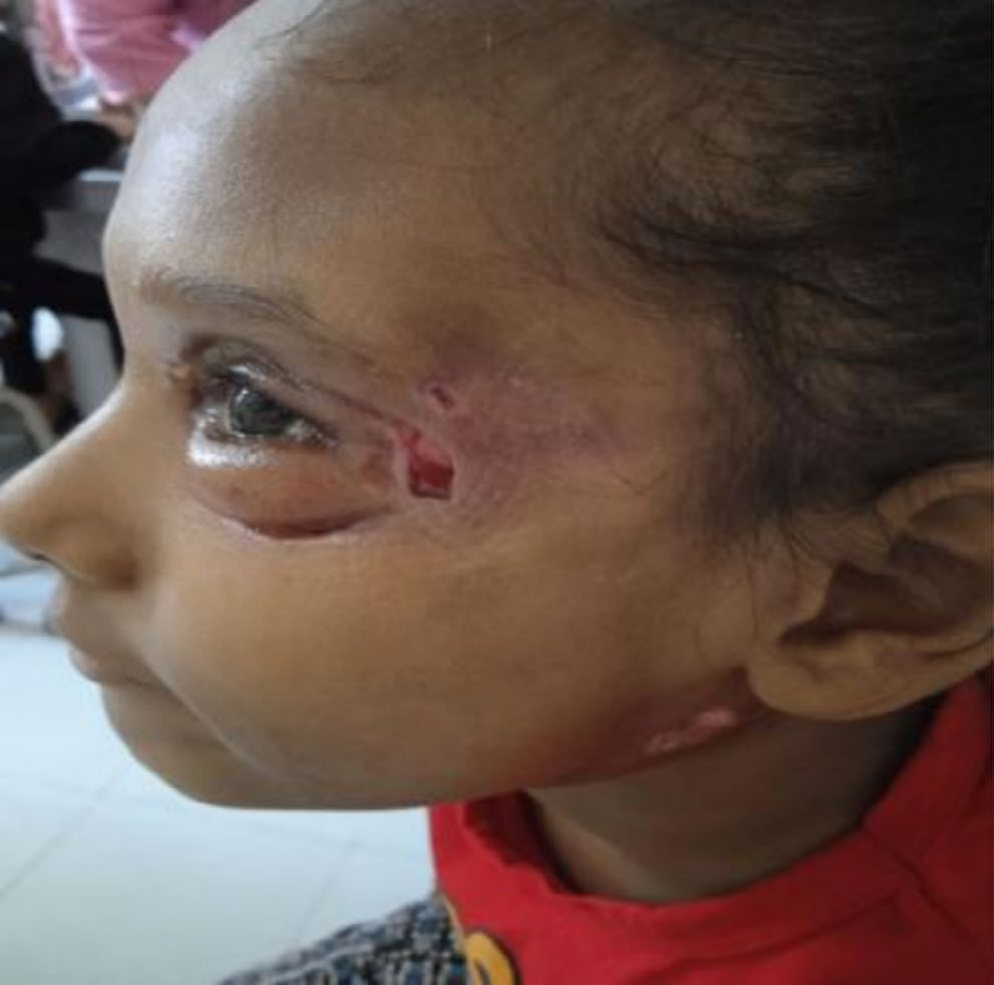
Clinical presentation showing pronounced erythema, swelling, and necrotic changes in both the upper and lower left eyelids, consistent with early necrotizing fasciitis.

**FIGURE 2:**
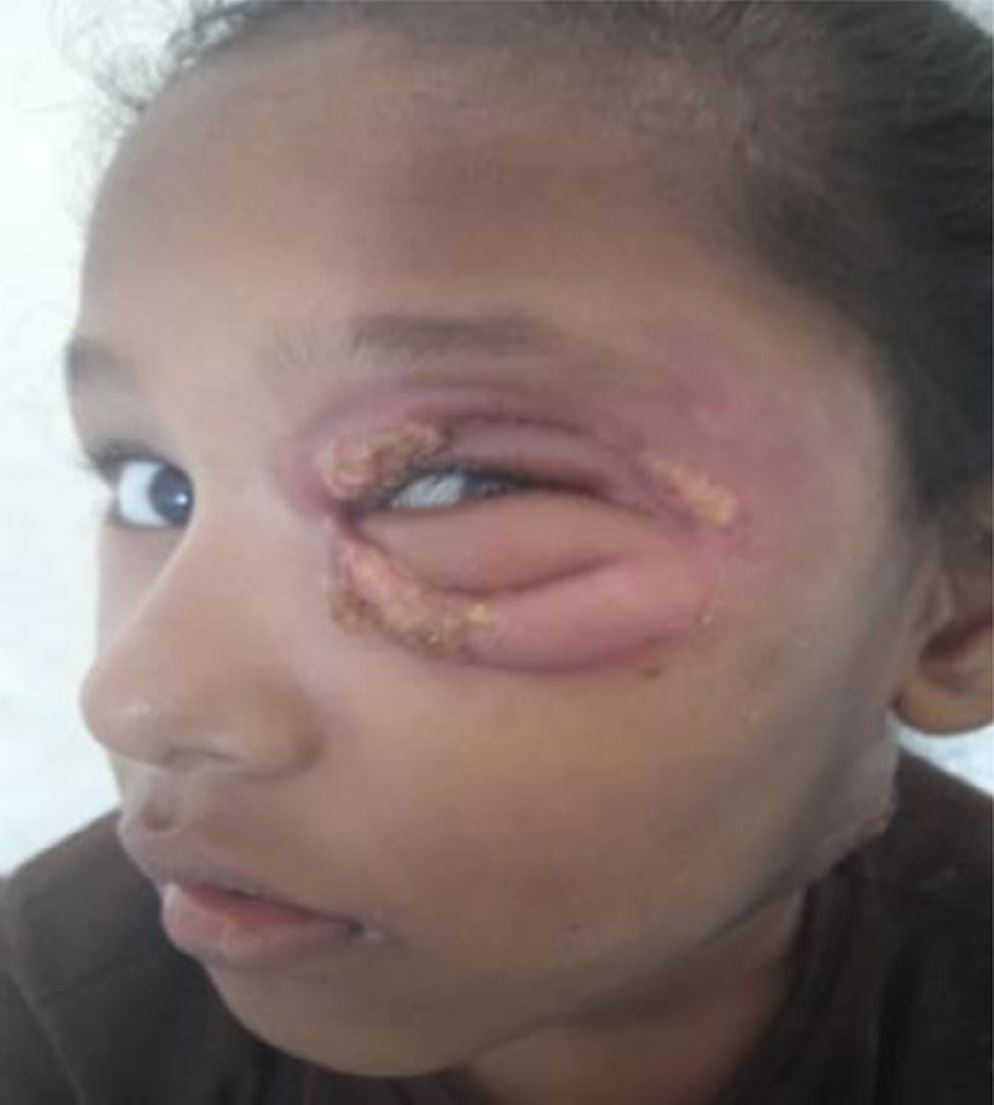
Clinical presentation showing pronounced erythema, swelling, and necrotic changes in both the upper and lower left eyelids, consistent with early necrotizing fasciitis.

Diagnosis of varicella was established clinically. She was admitted to the pediatric ICU and underwent incision and drainage. Upon presentation to the ophthalmology department, the patient exhibited necrosis of the periorbital skin and underlying tissue, along with erythematous swelling in both the left upper and lower eyelids (Figures 1 and 2).

## 3 Methods (Differential Diagnosis, Investigations, Management)

Differential diagnoses considered included drug reactions, monkeypox, mucormycosis, and bacterial NF. These were ruled out based on clinical presentation, non‐endemicity of monkeypox, and negative fungal tests. PCR testing for VZV, while standard, was unavailable; diagnosis was clinical. The diagnosis of necrotizing fasciitis of the left eye was established based on the development of a secondary skin infection following varicella eruptions, the presence of a necrotizing infection along the subcutaneous planes, and the absence of any inciting trauma, surgery, or insect bite. The swab from the conjunctiva and pus were sent for culture. The conjunctival swab was reported positive for CONS (Coagulase Negative Staphylococci), and pus was positive for Enterococcus. She received IV Meropenem and Vancomycin for 21 days. For swelling of the neck, ultrasonography of the head and neck, along with the jugular vein, was done, from which the diagnosis of Lemierre's syndrome was made. On excisional biopsy, it was negative for mycobacterium infection. The periorbital swelling was decreased, and the patient was improving, so she was discharged and was prescribed oral antibiotics (levofloxacin 250 mg, once a day for 2 weeks) and also called for follow‐up in 2 weeks.

But after 1 month, swelling over the eyelid worsened with associated peeling of the lid skin. There was no history of trauma or insect bite in the periorbital region during that course of time. The left periorbital skin exhibited significant necrotic changes, characterized by pronounced swelling, erythema, and induration of both the upper and lower eyelids. LRINEC score was calculated at 7, consistent with a high probability of NF. A distinct 2 × 2 cm patch of black necrotic tissue was observed on the lateral aspect of the left upper lid, accompanied by several small bullae containing serosanguinous fluid. The surrounding periorbital tissue showed diffuse swelling and redness, with notable tenderness on palpation. Crepitus was detected beneath the skin, suggesting potential underlying issues. Despite these concerning signs, visual acuity remained intact at 6/6 in both eyes, and extra‐ocular motility was fully preserved across all gazes. Examination of both the anterior and posterior segments revealed no abnormalities. Blood and pus culture from the affected area was sent, and wound exploration with left lower and upper eyelid debridement of necrotic tissue was planned under general anesthesia. Approximately 3 × 3 cm of necrotic tissue and slough, along with 2–3 mm of surrounding healthy tissue over the margin, was carefully debrided under aseptic conditions. The debrided tissue was sent for histopathological examination to determine the underlying cause and assess for any potential malignancy or infectious process. During aspiration, a dry tap was noted, indicating that there was likely no fluid collection or abscess present in the area. The pus was positive for *Acinetobacter calcoaceticus Baumannii complex*. Hence, treatment with intravenous flucloxacillin 550 mg and clindamycin 250 mg was initiated.

On the first postoperative day, the wound was assessed and found to be healthy, with no signs of discharge or necrotic tissue. Daily dressings were performed using normal saline, followed by a thorough wash with Gentamicin to maintain an optimal healing environment. Any necrotic tissue observed was promptly debrided under local anesthesia to promote healing.

## 4 Conclusions and Results (Outcome and Follow‐Up)

**FIGURE 3:**
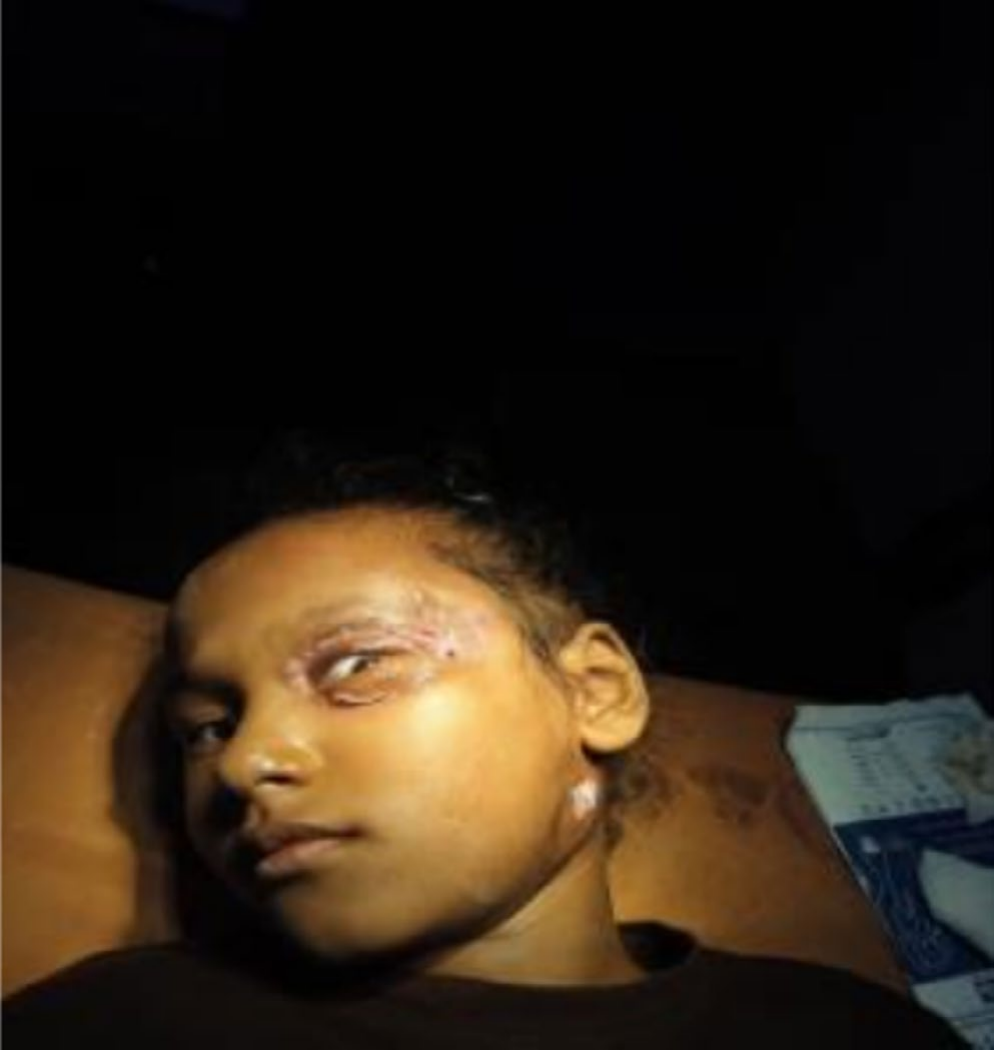
Postoperative image demonstrating healthy granulation tissue and progressive wound healing following surgical debridement and antibiotic therapy for periorbital necrotizing fasciitis.

On subsequent follow‐ups, the wound continued to show healthy granulation tissue, indicating good progress (Figure 3). As a result, the patient was discharged with oral antibiotics and specific care instructions. It's crucial for the patient to monitor the wound for any changes and adhere to follow‐up appointments to ensure ongoing recovery.

## 5 Discussion

Varicella is a highly infectious disease of children, which is usually mild in nature, without complications or sequelae. Occasionally, the virus of varicella may give rise to a number of complications of varying severity that may occur singly or in association, affecting the respiratory system, the nervous system, the urinary system, and the skin in descending order of frequency [9]. Necrotizing fasciitis is a very rare complication of varicella zoster virus. The rapid bacterial spread along the fascial plane characterizes NF as an infectious disease. It is a rare illness with an annual incidence of about 1.5 occurrences per 100,000 individuals; even the death rate is fairly significant, reaching 20%–30% [10]. This case is notable for the unusual progression from varicella to periorbital NF in a previously healthy child, as well as the timely use of surgical and antimicrobial therapies.

There are three distinct varieties of Varicella gangrenosa that have been described in literature: (A) moist gangrene, which is thought to be infective, most commonly caused by *hemolytic Streptococcus* or *Staphylococcus aureus* ; (B) dry gangrene, secondary to arterial thrombosis; (C) purpura fulminans, which is linked with disseminated intravascular coagulation (DIC) [11]. Varicella gangrenosa affects parts of the body like the trunk and extremities, but facial involvement is very rare due to robust vascularization. Most literature related to necrotising fasciitis in the periorbital region mentions trauma as the initial event leading to the disruption of the skin barrier. *Staphylococcus* and other bacteria have been isolated from cases of periorbital necrotising fasciitis [7]. It has also been suggested that the administration of nonsteroidal antiinflammatory drugs in chicken pox might enhance the development of necrotizing fasciitis [12, 13]. NF's defining feature is rapid bacterial spread along fascial planes, often requiring prompt surgical intervention. NSAIDs like ibuprofen can potentiate NF, though not used in our case. Acyclovir suppresses viral replication but does not clear latent virus. Timely initiation of acyclovir therapy can reduce complications; the recommended dose of acyclovir in an uncomplicated case is 800 mg five times a day for 7 days, while intravenous acyclovir is administered as 10 mg/kg for 7–10 days in complicated cases. Repeated debridements and intravenous antibiotics are important to prevent the spread of skin infections. Differentiation of this entity from mucormycosis is important in diabetic patients. Intravenous amphotericin is preferred if hyphal elements are found on sections of necrotic tissue [7, 13]. The WHO guidelines also propagate the use of intravenous acyclovir medications in varicella infection patients who are immunocompromised or severely complicated [8]. Our management included antimicrobials and surgical debridement. Histopathology ruled out fungal involvement.

The early surgical management of our case with eyelid and soft tissue debridement prevented ongoing damage to the vital eye structures. Poor penetration of antimicrobial medication into the areas of relative hypovascularity may explain the poor response of this infection to conventional intravenous antibiotic treatment, and therefore, aggressive surgical debridement is required. Earlier diagnosis and surgical therapy with prompt debridement can potentially lessen the extreme morbidity and potential mortality caused by Varicella gangrenosa [6].

Periorbital necrotizing fasciitis is a rare but severe complication of Varicella. Differentiating between necrotizing fasciitis and gangrene is essential. Our primary goals in managing this patient were to prevent intraorbital spreading of infection, preserve the sight, and restore the functions of the eyelid. Prompt surgical intervention helped to prevent further damage to critical eye structures and led to a positive outcome. This emphasizes the necessity for a multidisciplinary approach involving both the pediatric and ophthalmology teams in managing such complex cases.

## 6 Strengths and Limitations

This case is strengthened by its rarity, thorough diagnostic process, and multidisciplinary management. There was no feedback barrier between the investigator and the respondent because the study was conducted using a direct observational method. Limitations include an absence of PCR confirmation, a lack of detailed histopathology beyond fungal testing, and an unclear indication for corticosteroid use.

## Author Contributions

**Govinda Bhandari:** conceptualization, writing – original draft, writing – review and editing. **Himal Acharya:** formal analysis, project administration, writing – original draft. **Richa Paudyal:** supervision, writing – original draft.

## Ethics Statement

The authors have nothing to report.

## Consent

We confirm that written informed consent was obtained from the patient for publication of this case report and accompanying images.

## Conflicts of Interest

The authors declare no conflicts of interest.

## Data Availability

Data sharing does not apply to this article as no datasets were generated or analyzed during the current study.
